# Zeitlichkeit des Gegenwissens in der Ökologischen Landbau-Szene der Bundesrepublik (1970–1999): aus Alt mach Neu!

**DOI:** 10.1007/s00048-022-00351-w

**Published:** 2022-10-17

**Authors:** Alexander von Schwerin

**Affiliations:** Max-Planck-Institut für Wissenschaftsgeschichte, Berlin, Deutschland

**Keywords:** Gegenwissen, Umweltbewegung, ökologischer Landbau, Landwirtschaft, Bodenökologie, Konservative Modernisierung, Counter-knowledge, Environmental movement, Agriculture, Organic farming, (soil) ecology, Conservative modernization

## Abstract

Die Mitte der 1970er Jahre gegründete Stiftung Ökologischer Landbau (SÖL) trat an, den ökologischen Landbau in der Bundesrepublik zu fördern. Zu diesem Zweck führte sie Protagonisten aus Wissenschaft und Umweltbewegung zusammen, die unter Rückgriff auf die wissenschaftlich geprägten Konzepte des natürlichen und biologischen Landbaus der 1920er und 1930er Jahre eine Wissensgrundlage für den ökologischen Landbau aufbauen sollten. Anhand der Gründungsgeschichte, Struktur und Arbeit der SÖL lässt sich zeigen, dass Zeitlichkeit eine wesentliche Rolle bei der Etablierung alternativer Wissensbestände spielte. Entgegen dem etablierten Modell linearen wissenschaftlich-technologischen Fortschritts ging es um die Rückbesinnung auf Wissensbestände und Praktiken, die in vorhergegangenen Prozessen des Vergessens und der Marginalisierung aus dem wissenschaftlichen Wissenskanon, aber auch aus der landwirtschaftlichen Praxis verschwunden waren. Dies zeigt sich am Beispiel der sogenannten Spatendiagnose, einer in den 1930er Jahren von Bodenbiologen entwickelten Methode zur Beurteilung des Ackerbodens. Konzepte und Praxis des Gegenwissens liefen auf ein Modell konservativer Modernisierung im ökologischen Landbau hinaus.

## Abstract (en)

The Stiftung Ökologischer Landbau (SÖL), founded in the mid-1970s, set out to promote organic farming in the Federal Republic of Germany (FRG). To this end, it brought together protagonists from the scientific community and the environmental movement to build a knowledge base for organic agriculture by drawing on the science-based concepts of natural and organic farming of the 1920s and 1930s. Based on the history of its founding, its structure, and work, this article demonstrates that temporality played an essential role in the establishment of alternative bodies of knowledge. Contrary to the established model of linear scientific-technological progress, the aim was to return to bodies of knowledge and practices that had largely disappeared from the scientific canon of knowledge, but also from agricultural practice, in previous processes of forgetting and marginalization. This is exemplified by the so-called “spade diagnosis,” a method developed in the 1930s by soil biologists to assess arable soil. Concepts and practice of counter-knowledge amounted to a model of conservative modernization in organic farming.

## Einleitung: Zeit der Rückbesinnung

Im Jahr 1976 gründete das Industriellenehepaar Karl Werner Kieffer und Dagi Kieffer die Stiftung Ökologischer Landbau (SÖL) mit dem Zweck, Alternativen zur Intensivlandwirtschaft mit ihren verheerenden Folgen aufzuzeigen (Kieffer 1976). Diese Hoffnung sollte sich erfüllen. Mit ihren Publikationen, Veranstaltungen und ihrer Beratungstätigkeit erreichte die SÖL schon bald viele an Alternativen zur Intensivlandwirtschaft interessierte Menschen. Einen Höhepunkt ihres Einflusses bildete die von der SÖL betriebene Gründung des Verbandes Arbeitsgemeinschaft Ökologischer Forschungsinstitute (AGÖF) und der Zusammenschluss der warenzeichenverleihenden Verbände für Erzeugung, Verarbeitung und Vertrieb von Produkten ökologischer Landwirtschaft zum Superverband Arbeitsgemeinschaft Ökologischer Landbau in Deutschland (AGÖL) (Diel 1992: 152; von Ledebur 1992; Vogt 2000: 276).

In Gründung und Arbeit der SÖL manifestierte sich die in der Umweltbewegung verbreitete Infragestellung des herrschenden Landwirtschaftssystems und seiner wissenschaftlich-technischen Grundlagen. Die alarmierenden Nachrichten über die Bedrohung von Natur und Gesundheit sowie die wachsende Gewissheit von der Endlichkeit der Naturressourcen befeuerten ein grassierendes Krisenbewusstsein (Hünemörder 2004: 182–241; vgl. auch Radkau 2011: 148–152) und bestärkten das Misstrauen gegenüber den Fähigkeiten der etablierten Institutionen der Wissenschaft, Antworten auf die drängenden Fragen zu finden. Stattdessen setzte die Umweltbewegung auf ihre eigenen Potentiale und Möglichkeiten, dem Lernen aus der Praxis, experimentelle Projekte und gemeinschaftlich gemachte Erfahrungen.^1^

Die Rückbesinnung auf die eigenen Möglichkeiten hatte allerdings ihre Grenzen. Zu groß war die Wissenslücke, die zwischen dem verschmähten Heute der wissenschaftlich-technischen Welt und dem angestrebten Morgen einer alternativen Gesellschaft aufklaffte. Der alternative Wissenskorpus konnte sich also nicht im Modus des Präsentischen allein herstellen. Bereits der Soziologe Walter Hollstein machte in seiner Soziografie der sich formierenden „Gegengesellschaft“ die Rückbesinnung auf vergessene Erkenntnisse und Praktiken als eine der Quellen aus, aus denen die Alternativbewegung ihr Wissen schöpfte. Die ökologisch orientierte Landwirtschaft gehörte zu seinen Fallbeispielen:

Im Erlebnis der neuen Natürlichkeit werden auch alte und ältere Erfahrungen wieder aktualisiert: der biologisch-dynamische Landbau der Anthroposophen, die biologische Landwirtschaft englischer und skandinavischer Experten, der Saatkalender, kosmisches Denken, der Einfluß und die Bedeutung der Gestirne, Weisheiten der Naturvölker u. v. a. (Hollstein 1979: 129–130).

Die Rückbesinnung auf Entferntes und Vergangenes füllte die Lücke, die die Rückbesinnung auf die eigenen Möglichkeiten und Vermögen allein nicht zu füllen imstande war. Beides charakterisierte die Wissensbildung in der Alternativbewegung. Beträchtliche Unterschiede taten sich damit innerhalb des eh schon in Veränderung begriffenen Zeitgefühls der 1970er Jahre auf. Christina Brandt beschreibt dieses Zeitgefühl am Beispiel der Auseinandersetzung mit den heraufziehenden Biotechniken und in Anlehnung an Nowotny als „erstreckte Gegenwart“, in welcher die Zukunft ihren utopischen und offenen Charakter zugunsten eines technokratischen Modells verloren hat (Brandt 2010: 136–138).^2^ Dazu gehörte, dass auch das Management der Unzulänglichkeiten des technokratischen Modells, die umweltlichen Krisenerscheinungen der wissenschaftlich-technischen Welt und die Handlungszwänge neuer „Unübersichtlichkeiten“ (Habermas 1985; Faulenbach 2011) Politik, Wissenschaft und Öffentlichkeit zunehmend auf die Sicherung des Erreichten und Vorhandenen festlegten. Dagegen öffnete der von der Alternativbewegung eingeforderte Bruch mit der Gegenwart ungewohnte Zukunftsperspektiven.^3^ Die Ausgestaltung einer konkret-utopischen Zukunft der sogenannten Gegengesellschaft folgte allerdings nicht dem gewohnten Weg eines linear gedachten wissenschaftlich-technischen Fortschritts, sondern erfolgte im Rekurs und in Rückbesinnung auf andere Welten und Zeiten.

Zu denken ist dabei an theoretisches und praktisches Wissen, das aus dem Wissenskorpus der westlichen Naturwissenschaften herausgefallen oder erst gar nicht in jenen Eingang gefunden hatte: an nicht-westliche Wissenstraditionen, wie etwa die chinesische Medizin, die sich einer großen Aufmerksamkeit in der Alternativkultur erfreuten (Mende 2017: 183–184; Stadler et al. 2020: III/1–25), aber auch die Wiederentdeckung der eigenen, verschütteten Vergangenheit. Der einsame Chronist der Landwirtschaftswissenschaften in Deutschland Frank Uekötter spricht von „Prozessen des Vergessens“ und „Wissenserosion“, durch die in der Landwirtschaft der Bundesrepublik Wissensbestände der Vorkriegszeit verloren gegangen waren (Uekötter 2010: 319). Gemeint ist in diesem Fall ein durch den Wandel der Landwirtschaft sozial, industriell oder politisch verursachter Verlust, nicht die Abkehr von Wissensbeständen, die sich epistemisch als überholt erwiesen. Eine der Strategien der Umweltbewegung, Wissenslücken zu schließen, war mit anderen Worten, solches verlorenes Wissen zu heben – vergessene, marginalisierte und traditionelle Wissensbestände und Praktiken aus Europa und darüber hinaus aus anderen Ländern und Kulturen.

Das hier vorgestellte Beispiel der ökologisch orientierten Landwirtschaft spürt der Bedeutung und der Wiederentdeckung verdrängten Wissens in der ökologischen Bodenkunde nach. Die Vergangenheit spielte demnach eine entscheidende Rolle im ökologischen Landbau und zwar in mehrfacher Hinsicht: mit Blick auf Wissensinhalte und Praktiken, persönliche Erfahrungsräume und Netzwerke, das wissenschaftliche Selbstverständnis und die wissenschaftliche Praxis sowie den Fortschrittsbegriff. Anhand der in den 1930er Jahren entwickelten Spatenmethode lässt sich zeigen, welchen Stellenwert die Reaktualisierung von vermeintlich überholten Wissensbeständen für den ökologischen Landbau hatte. Die Reaktualisierung wendete diese althergebrachten Wissensbestände gegen die in den Hochschulen tonangebenden Landwirtschaftswissenschaften. Sie aktualisierte zugleich damit eine alte Entgegensetzung zwischen einer industriell und technisch ausgerichteten auf der einen sowie einer dem ganzheitlichen oder auch völkischen Natürlichkeitsdenken nahestehenden Landwirtschaft auf der anderen Seite, wie sie bis in die 1920er Jahre zurückreichte.

Allgemeiner gesprochen, kam in der Reutopisierung der Zukunft ein spezifischer Umgang mit gesellschaftlichen Veränderungen zum Ausdruck, der in der Alternativbewegung verbreitet gewesen zu sein scheint und der hier als konservative Modernisierung zusammengefasst wird.^4^ Eine ältere, durchaus etablierte Generation prägte diese spezifische Modernisierungsskepsis (vgl. Linse 1986; Graf 2008; Mende 2011). Die Erfahrungsräume und Wissensbestände, auf die diese Altvorderen der Umweltbewegung zurückgriffen, waren dabei von besonderer Bedeutung, da sie die Denkmuster und Handlungsoptionen der Alternativbewegung teilweise jedenfalls kanalisierten (vgl. auch Melzer 2003; Stoff 2015; Treitel 2017). Die reaktivierten Vergangenheiten schränkten den Horizont der gegengesellschaftlichen Utopien allerdings gleich wieder ein, wie das Beispiel der SÖL zeigt.

Die folgenden Abschnitte untersuchen die Struktur der Gründungsgeneration der SÖL, deren Einfluss auf die inhaltliche Ausrichtung, Strategie und Arbeit der Stiftung sowie die von der SÖL wieder aufgegriffenen Theorien und Praktiken der älteren Bodenkunde am Beispiel der sogenannten Spatendiagnose. Auch wenn an dieser Stelle offenbleiben muss, wie sich genau die bäuerliche Praxis gestaltete, so gibt die Renaissance dieses Verfahrens zur Bodenuntersuchung doch ein Beispiel für die Bildung und Zirkulation von theoretischem und praktischem Wissen an der Schnittfläche von Universität und Umweltbewegung.

## Öko-Krise, Stiftungs-Gründung und Altherren-Netzwerke

Die Anfang der 1970er Jahre ausgerufene „Öko-Krise“ fand schnell Niederschlag in unterschiedlichsten Aktivitäten zum Aufbau einer alternativen Gegenökonomie. Die Gruppierungen, Netzwerke und Milieus, die sich ausbildeten, können mit Blick auf ihre zum Teil länger zurückreichende Formierungsgeschichte unterschieden werden (Graf 2008: 213–222; Mende 2011). Das Alter der Protagonist*innen spielte dabei, wie in diesem Abschnitt zu sehen sein wird, eine bemerkenswerte Rolle. Motor der Entwicklung waren demnach gerade in den Anfangsjahren nicht nur pragmatisch gewendete Protagonist*innen der in Auflösung begriffenen Studentenbewegung oder die jungen Umweltbewegten. Die ‚gealterten Alternativen‘, die sich lange marginalisiert schon seit Jahrzehnten mit ökologischer Landwirtschaft befasst hatten, entstiegen nun ihren Zeitkapseln. Hinzu kamen die ‚gealterten neuen Alternativen‘, die sich erst im fortgeschrittenen Alter für die Sache der ökologischen Landwirtschaft einzusetzen begannen.

Dass die Frage nach Alt und Jung, Altem und Neuem unter der Oberfläche des aufkeimenden Alternativlebens virulent war, macht ein Blick auf eine der ersten Öko-Kommunen in Europa deutlich. Im Jahr 1972 verließen einige britische Familien ihr bürgerliches Zuhause, um auf einer verlassenen Farm im hügeligen Wales ein neues selbstgenügsames Leben zu erproben. Wer den Erfahrungsbericht eines der Mitgründer liest, erfährt, dass die Öko-Pionier*innen „self-sufficiency“ umfassend verstanden: vom Hausbau, über die Energieversorgung, den Haushalt, Gesundheit bis hin zu Landwirtschaft und Ernährung (Clarke 1977: 13–17). Entsprechend schnell stellten sich für BRAD – das Akronym für das als *Biotechnic Research and Development* bezeichnete Kommunenprojekt – fast unüberwindliche Schwierigkeiten ein. „Our generation may have lost the ancient crafts that used to be handed down from father to son“, resümierte der Autor Robin Clarke fast resignativ (Clarke 1977: 285). Der Faden der Tradition, der für die Weitergabe handwerklichen Wissens von einer Generation zu nächsten sorgte, war demnach abgerissen. Folgerichtig lautete die Devise, diesen abgerissenen Faden wiederaufzunehmen. In einem auch in der westdeutschen Umweltbewegung viel rezipierten und vielfach wiederabgedruckten Tableau, in dem Clarke die bestehenden und die anzustrebenden Verhältnisse gegenüberstellte, stand diese Aufgabe nicht zufällig an zentraler Stelle. Während die herrschende „harte technische Gesellschaft“ Junge und Alte voneinander entfremdete, würden diese in der künftigen „sanften technischen Gesellschaft“ wieder zusammenfinden (vgl. Stadler et al. 2020: V/47). Die darin formulierte Utopie der zukünftigen Gesellschaft huldigte nicht dem Jugendkult, sondern schöpfte aus dem Wissen und den Erfahrungen „der Alten“. Dass Clarke damit nicht nur ein Programm entworfen, sondern den Geist der Zeit getroffen hatte, zeigte sich nicht zuletzt in den Strukturen und der Praxis des ökologischen Landbaus.

Die Gründung der Stiftung Ökologischer Landbau (SÖL) im Jahr 1975 stellte auf dem Weg zum Ausbau der ökologisch orientieren Landwirtschaft in der Bundesrepublik einen Meilenstein dar (Schaumann et al. 2002: 85–114; Vogt 2000: 275).^5^ Im Zentrum der Gründung stand das Industriellenehepaar Karl Werner Kieffer und Dagi Kieffer. Karl Werner war nach langem Dienst als Vorstandsvorsitzender des Nähmaschinenunternehmens G. M. Pfaff AG in Kaiserslautern gerade in den Ruhestand getreten, als er sich zusammen mit seiner Frau Dagi entschloss, die gemeinnützige, wissenschaftsfördernde und zugleich praxisorientierte Stiftung ins Leben zu rufen (Kieffer 1982: 56–57). Dagi, die aus dem pfälzischen Weinbau stammte und deshalb mit den Problemen der Landwirtschaft vertraut war, übernahm die Geschäfte der SÖL (Kieffer 1992: 175). Der Wirtschaftsingenieur Karl Werner konzentrierte seine Kräfte auf die zeitgleich gegründete Stiftung Mittlere Technologie, die seinen ökonomischen Kenntnissen und Interessen an technologisch-industrieller Entwicklung eher entsprach (hier und nachfolgend Kieffer 1982: 36–61).

Die Geschehnisse rund um das geplante Atomkraftwerk Wyhl in der Rheinebene bei Freiburg gaben den Anlass zur Gründung der Doppelstiftung. Die grundsätzliche Bereitschaft und erste Ideen waren indes bereits zuvor gereift, unter anderem durch das Engagement von Dagi in der Anti-Atomkraft-Bewegung und in der Auseinandersetzung mit dem zweiten, im Jahr 1974 erschienenen Bericht des *Club of Rome* unter dem Titel *Menschheit am Wendepunkt* sowie den Thesen des deutschstämmigen britischen Wirtschaftsberaters Ernst F. Schumacher, Autor des einflussreichen Buches *Small is beautiful. A Study of Economics As If People Mattered* (1973). Der Einfluss dieser internationalen Denkanstöße war bei der Gründung unübersehbar. Die ökonomisch-strukturellen Lösungsvorschläge des *Club of Rome* fanden sich im Stiftungsauftrag wieder. Viel mehr noch aber fühlten sich die Kieffers von den Postulaten Schumachers angesprochen, der die Lösungsvorschläge des *Club of Rome* in bezeichnender Weise kritisierte:

Wie angenehm und ermutigend wäre es gewesen, hätte man in diesem Bericht von über zweihundert Seiten auch nur an einer einzigen Stelle eine Rückbesinnung auf den Menschen und seine eigentlichen Bedürfnisse und Anliegen gefunden; oder eine kleine Einsicht, daß es möglich sei, die Technik dem Menschen anzupassen, anstelle der derzeitig erzwungenen Anpassung des Menschen an die Technik […] oder ein paar Hinweise auf die Experimente, die an vielen Stellen der Welt bereits im Gang sind, wo junge und nicht mehr so junge Menschen konkret und frohgemut an der Herausbildung eines neuen, gesunden, auf Dauer und Frieden abgestellten Lebensstils arbeiten (Schumacher 1974: 131–133; vgl. Kieffer 1982: 41).

Die von Schumacher beschworene Rückbesinnung auf das „menschliche Maß“ zielte letztlich auf Versöhnung: die des Menschen mit der technischen Entwicklung, mit der Natur, mit sich selbst und nicht zuletzt der Generationen (vgl. Schumacher 1974: 20, 49, 52). Die Perspektive, die er damit aufmachte, fand enthusiastische Aufnahme bei vielen Zweifelnden und Suchenden und regte verschiedentlich vor allem auf Entwicklungsarbeit ausgerichtete Projekte nach dem Vorbild Schumachers an (Schumacher 2011). Schumacher hatte lange erfolgreich in der Wirtschaft gearbeitet, bevor er 1966 mit der *Intermediate Technology Development Group* (ITDG) eine Organisation gründete, die Entwicklungs- und Anwendungsperspektiven für Technologien verbreiten sollte, welche speziell an die lokalen Bedingungen und Bedürfnisse in den Entwicklungsländern angepasst waren. Daneben engagierte er sich für den ökologischen Landbau, weil beides in seinem Verständnis in engem Zusammenhang zueinanderstand (Schumacher 1974: 50–51, 192; Moss 2013: 16–20; Radkau 2011: 264). Auch die Kieffers sahen sich von Schumachers Vorbild ermutigt. Bei einem gemeinsamen Treffen in Kaiserslautern schlug Schumacher vor, etwas Ähnliches in Deutschland aufzubauen und dabei alternative Technologie-Entwicklung und ökologischen Landbau miteinander zu verzahnen (Kieffer 1982: 42; Kieffer 1992: 175). Das Alter der Protagonisten spielte bei diesem Ideentransfer bereits eine Rolle. Der Umstand, dass Schumacher wie er selbst um die sechzig Jahre alt war, half Karl Werner ebenso sehr, das nötige Vertrauen zu fassen, wie die gemeinsame Fachsprache, die „Sprache des Managers“ (Kieffer 1982: 45).

Mit der Doppelstiftung SÖL und Mittlere Technologie machte sich eine ältere Generation die Entwicklung der ökologischen Landwirtschaft zur Aufgabe. Deutlich wird dies anhand des Personenkreises, den die Kieffers für die SÖL verschiedentlich mobilisierten. Da waren zum einen jene fachkundigen Sympathisanten und Interessierte, die die Kieffers im Jahr 1976 zusammenriefen, um Vorschläge für künftige Leitlinien, Strategien und Arbeitsschwerpunkte der SÖL zu erörtern (Stiftung Ökologischer Landbau 1976: 9–10). Das Durchschnittsalter der Vertreter aus Politik, Bürgerinitiativen und Wirtschaft lag bei 42 Jahren.^6^ Der jüngste Teilnehmer war der Student Dieter Teufel als Vertreter des Bundesverbandes Bürgerinitiativen Umweltschutz (BBU), gefolgt vom Erben der Eigentümerfamilie des Saatgutkonzerns KWS AG Andreas Büchting mit 29 Jahren. Herausstachen zudem der mit 36 Jahren außerordentliche junge Präsident der Gesamthochschule Kassel Ernst Ulrich von Weizsäcker und der 39-jährige umweltbewegte Theologieprofessor Günter Altner (vgl. Weizsäcker 2014; Schmidt 2022).

Beim engeren Beraterkreis setzten die Kieffers sehr viel mehr auf Berufserfahrung und gesellschaftlichen Einfluss. Für die Mitglieder des Beirats der SÖL gewannen sie etablierte und namhafte Wissenschaftler sowie mit dem ehemaligen Kommissionspräsidenten der EWG Sicco L. Mansholt auch einen namhaften Politiker – ehemals berüchtigt für seine Großagrarpolitik, nun aber geläuterter Vorzeige-Sympathisant alternativer Bestrebungen (Dahl et al. 1976: 20). Das durchschnittliche Alter der elfköpfigen Herrenrunde betrug im Gründungsjahr der SÖL rund 58 Jahre.^7^

Im Stiftungs-Kuratorium schließlich versammelten die Kieffers vor allem freundschaftlich verbundene Personen und Praktiker mit einem Altersdurchschnitt von rund 57 Jahren:

- Da war etwa der 1913 geborene Thorwald Risler, der als Generalsekretär des Stifterverbandes für die deutsche Wissenschaft die SÖL und die Stiftung Mittlere Technologie beriet und treuhänderisch verwaltete (Kieffer 1982: 75; vgl. Sattler 2016: 603).
- Der Landwirt Gotthilf M. Goyert (*1922) aus Carlsberg bewirtschaftete seit den 1960er Jahren den ökologisch ausgerichteten „Neuhof“ (Karutz o.J.).
- Der Landmaschinenfabrikant Ernst Weichel (*1922) aus Heiningen prägte in den 1970er Jahren die Entwicklung der organisch-biologischen Bodenbewirtschaftung durch sein Engagement und die Entwicklung neuer Bodenbearbeitungsgeräte (Vogt 2000: 234; Anonym 2008: 471).
- Der promovierte Landwirt Erich Siefert (*1914) verfügte seit Anfang der 1940er Jahre über Erfahrungen in der Landwirtschaftsverwaltung und leitete den Arbeitskreis Naturgemäßer Landbau in Einbeck-Salzderhelden (Siefert 1943).
- Der Biologe Dr. Michael Lohmann (*1933) von der Werkhof Gemeinschaft e. V. in Egglham betreute im Hanser-Verlag die Reihe *Umweltforschung* und betätigte sich als gern gelesener Sachbuchautor (Hohlt 2014).
- Und schließlich der Agrarwissenschaftler Gerhardt Preuschen (*1908), der aufgrund seiner großen Erfahrung in der landwirtschaftlichen Beratung zu einem Hauptakteur in der Arbeit der SÖL werden sollte (Lünzer 1992: 373–374).

Die Mitglieder dieser um die Leiterin der SÖL Dagi Kieffer gruppierten Herrenriege fungierten als die wesentlichen Stichwortgeber in den Anfangsjahren der SÖL und gehörten zum Teil zum ideologischen Kern der sogenannten Öko-Konservativen, die in der Tradition konservativer Technik- und Fortschrittskritik standen. Das Milieu, das sich in der Umweltbewegung und speziell auch im ökologischen Landbau zusammenfand, war bekanntlich höchst divers und reichte von traditionell, bildungsbürgerlich und kulturkonservativ geprägten Naturschutzvereinen bis zu links-alternativen Umweltgruppen (Melzer 2003; Fritzen 2006; Engels 2010: 412–413; Treitel 2017: 265–280). Rüdiger Graf konstatiert sogar eine „konservative Tendenzwende“ in der Umweltbewegung, die maßgeblich auf den Einfluss einer älteren, Technologie-kritischen Generation, die „Öko-Konservativen“, zurückging (Graf 2008: 222–224). Der gedankliche und ideologische Kern der Öko-Konservativen kristallisierte sich in den 1970er Jahren vor allem im Umfeld der 1971 gegründeten Zeitschrift *Scheidewege* heraus (vgl. auch Mende 2011: 298–304). Karl Kieffer war ein großer Bewunderer des mit ihm befreundeten Unternehmers und Herausgebers der *Scheidewege* Max Himmelheber (Kieffer 1982: 44–45). Im sogenannten *Bussauer Manifest* brandmarkten Himmelheber, die Beirats- und Kuratoriums-Mitglieder der SÖL Schwabe und Lohmann, der *Zeit*-Autor Jürgen Dahl (*1929) sowie der ehemalige Leiter der Bundesanstalt für Naturschutz Gert Kragh (*1911) das durch die technische Zivilisation bedingte Missverhältnis zwischen Mensch und Natur als eigentliche Ursache des aus dem Ruder gelaufenen Wachstums.^8^ Die *Scheideweg*-Gruppe publizierte verschiedentlich auch in der Halbjahresschrift der Kieffer’schen Stiftungen *Bildung und Gesundheit*, so auch das besagte *Bussauer Manifest* (vgl. Dahl et al. 1976).

Das Netzwerk der SÖL war also weitgespannt und vom Stifterehepaar gezielt zusammengestellt; nicht nur mit Blick auf eine biografisch verbürgte ideologische Authentizität, sondern auch auf die Notwendigkeiten der anstehenden Stiftungsarbeit. Der Beirat bestand fast durchweg aus Wissenschaftlern und Universitätsprofessoren. Gefragt waren aber auch Praktiker, die die Kieffers wiederum vor allem im Kuratorium um sich scharten: Landwirte, Publizisten und Multiplikatoren, Unternehmer und Berater. Auch in dieser Hinsicht war die Altersstruktur dieser Gremien nicht zufällig. Denn Karl Kieffer war überzeugt, dass diejenigen „aus der Generation der über 60jährigen“ ihre „großen Erfahrungen in der Zeit nach der Pensionierung […] bei der Förderung des Neuen“ besonders gut nutzen konnten (Kieffer 1982: 77). Dies war also die Mischung, mit der der ökologische Landbau in der Bundesrepublik auf den Weg gebracht werden sollte.

## Wissenskrise, Rückbesinnung und Beratung im ökologischen Landbau

Wissen mobilisieren, verbreiten und in der Praxis implementieren – so kann man die Arbeitsfelder zusammenfassen, die die Stiftung Ökologischer Landbau (SÖL) bei der Förderung des ökologischen Landbaus Mitte der 1970er Jahre anging. Die Geschäftsstelle der SÖL, die 1976 mit bescheidener Ausstattung ihre Arbeit in Kaiserslautern aufnahm, war mit der Organisation dieser Aufgaben betraut.^9^ Der Bedarf an alternativen Informationen und praxistauglicher Beratung war auf dem Weg zu einer ökologisch basierten Landwirtschaft groß. In der Alternativszene kursierten Berichte aus Landkommunen, die konkret und anschaulich über eigene Erfahrungen mit ökologischer Landwirtschaft berichteten, sei es über energetische Stallbelüftung, Düngerverwertung oder Wärmegewinnung aus Kuhdung (Hollstein 1979: 126). Entgegen solchen idiosynkratischen Ansätzen musste überhaupt erst das Bewusstsein dafür geschaffen werden, dass die Geschichte ökologisch ausgerichteter Landbaumethoden bis in die 1920er Jahre zurückreichte und einen reichlichen Erfahrungsschatz parat hielt (Vogt 2000: 24).^10^ In einem SÖL-Text hieß es etwa:

Was liegt näher, als nach Alternativen zu suchen […] Solche Alternativformen des Landbaus gibt es. Es sind nicht nur die Formen, die bis kurz vor dem 2. Weltkrieg besonders in Deutschland zu sehr hoher Blüte geführt worden sind, sondern auch in anderen Ländern […] Das Wissen darum ist sehr verstreut. Die Kenntnis der Durchführung, eine für den Landwirt ja entscheidende Voraussetzung, ist vielfach verloren gegangen (Preuschen 1976: 40–41).

Aus der Erkenntnis, dass wertvolles Wissen in Vergessenheit geraten war, ergaben sich die konkreten Aufgaben der Stiftung. Die nützlichen Erkenntnisse der Vergangenheit mussten wiederentdeckt, Alt und Neu kombiniert sowie an Landmann und Landfrau gebracht werden. Tatsächlich konzentrierte sich die SÖL in ihren Anfangsjahren auf die Aufgaben einer solchen Rückbesinnungsarbeit – daneben trat mit den Jahren verstärkt auch die politische Lobbyarbeit auf Ebene der Verbände und Institutionen (Schaumann et al. 2002: 94–96).

Zunächst ging es darum, einen systematischen Überblick zu erarbeiten: „zu sammeln, was an Erfahrungen in den verschiedenen biologischen Richtungen“ bereits vorhanden war, um auf dieser Grundlage zu beurteilen, wo noch Entwicklungsarbeit geleistet werden musste (Preuschen 2002: 343). Die Dokumentation der vorhandenen Literatur war der erste Schritt. Im Archiv und der Bibliothek der Stiftung sammelten die Mitarbeiter*innen Bücher, Zeitschriften, Foto‑, Dia‑, Tonband- und Videomaterial, später auch in Zusammenarbeit mit der Zentralstelle für Agrardokumentation und Information in Bonn (Schaumann et al. 2002: 9). Daneben sollten die wenigen, noch lebenden Pioniere des ökologischen Landbaus, die bis dahin „auf verlorenem Posten“ geblieben waren, ins Gespräch gebracht werden (Preuschen 1976: 42–43). Als Ergebnis der vorläufigen Bestandsaufnahme erschien im Jahr 1977 ein erster *Leitfaden für den ökologischen Landbau* und zwar im österreichischen Stocker-Verlag, welcher bereits in den 1930er Jahren zu diesen Themen publiziert hatte (Stiftung Ökologischer Landbau 1977; Preuschen 2002: 344–345). Der fortschreitende Erkenntnisprozess in der Zentrale schlug sich in einer stetigen Ausdehnung der Publikationstätigkeit der SÖL nieder. Alte, reaktivierte und neue Erkenntnisse flossen gleichwertig in die zahlreichen allgemeinen und speziellen Publikationen ein, mit Titeln wie *Praxis des Öko-Anbaus, Die Ackerbaulehre nach ökologischen Gesetzen, Ökologischer Feldgemüseanbau, Nichtchemische Unkrautregulierung, EDV-Programm zur Planung und Analyse ökologisch wirtschaftender Betriebe* (Schaumann et al. 2002: 96). Eine Reihe von Publikationsserien, mit denen die SÖL unterschiedliche Klientel ansprach, kamen hinzu.^11^

Die regelmäßig herausgegebenen *Umstellungshefte* und *Beraterrundbriefe* signalisierten bereits im Titel, dass es der SÖL nicht bloß um die Vermittlung von Informationen ging, sondern um Beratung. Die Stiftung setzte damit an entscheidender Stelle an, war doch das landwirtschaftliche Beratungswesen seit jeher von großer Bedeutung für die landwirtschaftliche Praxis (Rid 1984: 328; Uekötter 2010: 332–338). Die ersten Praxistests bestätigten die Erwartung, dass die Umstellung der Bewirtschaftung eines Hofes auf ökologischen Landbau ohne langfristige beratende Begleitung kaum zu schaffen war (Preuschen 2002: 350).^12^ Die Schlussfolgerung daraus war, dass „Umfang und Qualität von Beratung für die weitere Ausbreitung des ökologischen Landbaus eine Schlüsselstelle“ einnehmen würden (Vogt 2000: 277). Die SÖL nahm deshalb gezielt die Weiterbildung der etablierten und nachrückenden landwirtschaftlichen Beraterinnen und Berater in Angriff, damit diese die Betriebe durch die Vermittlung grundlegenden Wissens, sorgfältige Planung in der Umstellungsphase und sachgerechte Ratschläge in kritischen Situationen unterstützen konnten (Preuschen 2002: 343). Im Februar 1977 veranstaltete die SÖL die erste Fortbildungsveranstaltung für landwirtschaftliche Beratungskräfte. Seitdem stand die Aus- und Fortbildung der „Multiplikatoren auf unterer Ebene“ mit im Mittelpunkt der Stiftungsaktivitäten (Geschäftsführung 1977: 40–41). Ein weiteres Erfolgsgeheimnis lautete, dörfliche Stammtische zu initiieren und einen „überzeugten Landwirt als Anführer“ zu gewinnen (hier und nachfolgend Preuschen 2002: 348–350). Mit Hilfe von Pfarrern und zuständigen Landwirtschaftsräten führte die SÖL vor Ort in Dorfkneipen, Pfarrhäusern und Versammlungssälen Informationsveranstaltungen durch. Auf diese Weise initiierte Stammtische perpetuierten die Diskussion und den Austausch. Der Stammtisch in Neumarkt bei Regensburg etwa arbeitete unter Führung eines Landwirts über Jahre selbständig und kümmerte sich gleich auch um ökologischen Biernachschub; jedenfalls ging das ökologisch gebraute Neumarkter Lammsbräu aus diesem Zusammenhang hervor.

Der Hauptverantwortliche für Beratungsfragen und zugleich „wissenschaftlicher Betreuer der Stiftung“ war der 1908 geborene Gerhardt Preuschen (Preuschen 1976: 41). Mit ihm hatten Dagi und Karl Kieffer einen Glücksgriff gelandet. Preuschen war nicht nur ein überaus charismatischer Redner; mit jahrzehntelanger Berufserfahrung als Berater und als emeritierter Direktor des Max-Planck-Instituts für Landarbeit und Landtechnik (MPI) im nahen Bad Kreuznach war er vom Fach (vgl. Preuschen 2002). Preuschen gehörte damit auch zu jener älteren Generation von Ökoaktivisten in Schlips und Kragen, die durch Ausbildung in der Weimarer Republik und Berufserfahrung in der Zeit des Nationalsozialismus geprägt waren (vgl. Uekötter 2010: 419). In seinem neuen Arbeitsumfeld der SÖL musste er sich nur wenig umstellen. Als rechte Hand von Stifterin Kieffer war Preuschen organisatorisch und lobbyistisch im Namen des ökologischen Landbaus in den ihm vertrauten Netzwerken der Landwirtschaftspolitik unterwegs. Denn entsprechend seiner Prägung als Landbauexperte, der seine Forschung und Expertise im Nationalsozialismus und in der Bundesrepublik immer in den Dienst des Staates gestellt hatte, setzte er weniger auf die Kräfte der Graswurzelbewegung (vgl. Heim 2003: 91–102; Raehlmann 2005: 123–128; Schwerin: in Vorbereitung), sondern auf die staatliche Verantwortung, Ökologie und Umweltschutz zu einer staatlich organisierten Erziehungsaufgabe zu machen.^13^ Versuche, den ökologischen Landbau in der landwirtschaftlichen Offizialberatung zu verankern, blieben aber ein mühsames Unterfangen (Vogt 2000: 276).

Die ausgerufene Umweltkrise und der Beginn des Umweltzeitalters in der Bundesrepublik waren auch für Agrarwissenschaftler*innen wie Preuschen eine Art Weckruf (hier und nachfolgend: Schwerin: in Vorbereitung). Schon als Max-Planck-Direktor hatte sich Preuschen kritisch mit der Landwirtschaftspolitik auseinandergesetzt, vor allem hinsichtlich ihrer Auswirkungen auf die traditionellen ländlichen Strukturen. Sein Institut galt nicht zuletzt deshalb innerhalb der Max-Planck-Gesellschaft (MPG) als Außenseiter. Viele der Naturwissenschaftler*innen der MPG hielten die am MPI in Bad Kreuznach betriebene traditionelle, am Feldversuch und ökonomisch orientierte Wissenschaft für zu wenig naturwissenschaftlich ausgerichtet. Die ganzheitlich und praxisorientierte Forschung Preuschens, die die bäuerliche Arbeit im Gesamtzusammenhang von Ertragssteigerung, Existenzsicherung und nachhaltiger Bodenpflege untersuchte, stand im Gegensatz zum übermächtigen Trend innerhalb der Biowissenschaften in Richtung experimenteller Laborforschung. Diese Spannung führte innerhalb der MPG zu Verteilungskämpfen mit dem Ergebnis, dass Preuschens MPI auf dem Altar der Molekularisierung der Lebenswissenschaften geopfert wurde. Mitte der 1970er Jahre schloss die MPG im Zuge größerer Umstrukturierungen und anlässlich der Emeritierung Preuschens die Pforten des MPI in Bad Kreuznach.

Emeritierung und Austritt aus der akademischen Gemeinschaft wirkten auf Preuschen wie ein Befreiungsschlag. Befreit von den Abhängigkeiten innerhalb der MPG und von der Industrie, radikalisierten sich Preuschens Ansichten, quasi parallel zur Radikalisierung der Umweltbewegung (vgl. Preuschen 2002: 377). In deutlichen Worten prangerte der bald als „zorniger alter Mann der Agrarwissenschaften“ oder „Rebell“ mit „kämpferischem Naturell“ apostrophierte Preuschen die „Verbrechen an der Fruchtbarkeit unserer Erde“ und damit die ökologischen und nicht zuletzt gesellschaftlich zerstörerischen Folgen der wirtschaftlichen und sozialen Modernisierungsdynamik auf dem Lande an (Preuschen 1973: 269; Preuschen 1977: 19). Die „Maßlosigkeit unserer Industriegesellschaft“, so schrieb er an den Präsidenten der MPG, der ungebrochene Fortschrittsglaube, die naturwissenschaftlich geprägte Intensivlandwirtschaft und die sogenannte Grüne Revolution stellten nichts weniger aufs Spiel als die landwirtschaftlichen Grundlagen der Gesellschaft. Die Landwirte und Landwirtinnen seien in zunehmende Abhängigkeit von Landmaschinen- und Saatgutbetrieben, der Düngemittelfirmen und vor allem der Chemieindustrie geraten, weshalb bei der rasant zunehmenden Technisierung in der Landwirtschaft Nutzen und Effektivität der neuen Technologien oftmals gar nicht mehr hinterfragt würden (Preuschen 1977: 17–20; Preuschen 2002: 346–347, 377):

Während in früheren Zeiten die Landwirte über lange Zeit in gleicher Art wirtschaften konnten, werden sie heute fast täglich mit neuen Technologien, Arbeitsverfahren, Maschinen und anderen Betriebsmitteln konfrontiert. Anzeigen, als Fachartikel getarnte Anzeigen im Textteil, reich bebilderte Schriften der Hersteller von Betriebsmitteln versuchen dem Landwirt die Notwendigkeit aller dieser oft kostspieligen Anschaffungen schmackhaft zu machen. Alle Empfehlungen laufen darauf hinaus, daß der Betriebsertrag erhöht und damit das Einkommen gesteigert würde. Wie aber soll der Landwirt kontrollieren, ob die versprochenen Erfolge wirklich eingetreten sind (Preuschen 1981: 1)?

Damit deutete sich bereits die Wissenskrise an, die die Macher*innen der SÖL als Kern der durch die naturwissenschaftlich-technische Modernisierung fehlgeleiteten Prozesse in der Agrarproduktion ausmachten, aber auch als Ansatzpunkt für die Lösung. Stiftungsgründer Kieffer beklagte, dass das dominierende Fortschrittsdenken nur den „Durchbruch nach vorne“ kannte (Kieffer 1982: 162). Die von den Naturwissenschaften ermöglichte Intensivlandwirtschaft und die von ihr mit vorangetriebene „Übertechnisierung“ (Schaumann et al. 2002: 79) förderte demnach die Dominanz der Agrarindustrie auf den Höfen, in Folge derer die Landwirte und Landwirtinnen das Wesentliche aus den Augen verlören und altes, bewährtes Wissen zunehmend verloren ging. „Zu keiner Zeit“, beschwor Preuschen die Missstände, „ist die Mißachtung des Humus so groß geworden wie in den letzten 20 Jahren in Europa, ausgerechnet da, wo die Heimat einer vernünftigen Humuswirtschaft bisher zu finden war“ (Preuschen 1977: 19). Manche Landwirte hätten im Zuge des wirtschaftlichen Existenzkampfes vergessen, auf eine nachhaltige und naturnahe Bodenbewirtschaftung zu achten, mit fatalen Folgen auf Dauer.

Solche Art Skepsis gegenüber dem naturwissenschaftlichen Kausaldenken und technizistischen Fortschrittsdenken musste die SÖL nicht neu erfinden. Die Kritik am Atomismus moderner Naturwissenschaften, die mit dem Lob von Ganzheitlichkeit und Natürlichkeit einherging, war in der im Umfeld der Lebensreform und rechtsgerichteter zivilisationskritischer Strömungen der 1920er und 1930er Jahren geprägten Generation von Naturforscher*innen und Mediziner*innen verbreitet (siehe z. B. Potthast 2001; Harrington 2002; Stoff 2012: 149–175, 286–287). Preuschen hatte ein Leben lang mit solchen Ansichten sympathisiert und ist insofern einmal mehr ein Beispiel dafür, dass die in den 1960er Jahren eingesetzte Technologiedebatte keineswegs nur das Anliegen von Soziolog*innen und Intellektuellen der jungen Bundesrepublik war.^14^

Expertise und Modernisierungskritik Preuschens schienen wie gemacht, um Antworten auf die drängenden Fragen der Zeit zu geben.^15^ Wenn das naturwissenschaftliche Wissensregime wichtige Wissensbestände verdrängte und das Grundübel der Entfremdung von der traditionellen Landwirtschaft und ihren naturnahen Arbeitsformen noch beförderte, musste Rückbesinnung auf die wertvollen, verloren gegangenen Wissensbestände die Antwort sein (vgl. Preuschen 1973: 269; Vogt 2000: 285). In den Worten Preuschens: „Hierfür müssen wir uns an Dinge erinnern, die früher so selbstverständlich waren, daß man kaum darüber sprach“, die in der heutigen Zeit aber „fast in Vergessenheit geraten sind“ (Preuschen 1981: 1). Den gescheiterten Naturwissenschaften setzte der Agrarexperte der SÖL die „Liebe und Ehrfurcht“ vor der „Mutter Erde“ entgegen (Preuschen 1985). Solche Worte bedienten zwar einen vor allem im Umfeld der Ökobewegung nicht selten anzutreffenden Agrarromantizismus, werbewirksam strich die SÖL indes heraus, dass ihr wissenschaftlicher Berater aus seinem reichen Erfahrungsschatz in differenzierter Weise zu schöpfen wusste:

Aus den Erfahrungen der Vergangenheit und aus den Erkenntnissen einer neuen Naturbetrachtung entwickelt der Verfasser eine neue Form der Bodennutzung in Zusammenarbeit mit der Natur und unter Beachtung ihrer Gesetze. Gestützt auf die Erfahrung eines achtzigjährigen Lebens im Dienste der Landwirtschaft, zeigt er die praktischen Wege, die Angst zu nehmen und Hoffnung zu wecken (Preuschen 1988: Klappentext).

In diesen programmatischen Sätzen klang an, dass die SÖL nicht einfach die althergebrachte traditionelle Landwirtschaft zum Rettungsmodell für die Agrar- und Umweltkrise ausrief, sondern für eine differenzierte Praxis der Rückbesinnung stand.

## Aktualität und Zukunftsfähigkeit alten Wissens: die Spatendiagnostik

„Wir wollten – angesichts mancher spöttischer Bemerkungen über eine ‚steinzeitliche‘ Technik – nicht als Außenseiter oder ‚Spinner‘ abgestempelt werden“ (Kieffer 1982: 48). So oder so ähnlich formulierte nicht nur Stiftungsgründer Kieffer die Probleme, die man sich mit dem Lob der Vergangenheit aufgeladen hatte (vgl. Schumacher 1974: 205–206; Treitel 2017: 170–171). Immer wieder musste man Unterstellungen entgegentreten, die den alternativen Projekten Rückwärtsgewandtheit, Wissenschaftsfeindlichkeit oder gleich den Rückfall in die Steinzeit vorwarfen. Die SÖL verabsolutierte dagegen nicht die Reaktivierung und Bewahrung verdrängter und vergessener Wissensbestände, Praktiken und Erfahrungen und lehnte die technische Modernisierung nicht rundweg ab. Die SÖL förderte den ökologischen Landbau deshalb auch bewusst in Abgrenzung und als Alternative zu anderen Strömungen, insbesondere zu den erfolgreichen Netzwerken des biologisch-dynamischen Landbaus und seinem anthroposophischen Natur- und Menschenbild (vgl. Vogt 2000: 261).

Gegen Ende der 1970er Jahre begann die SÖL mit der sogenannten Spatendiagnostik ein altes, wiederentdecktes Verfahren zu propagieren. Ähnliche Ansinnen vertrat etwa in Österreich die Förderungsgemeinschaft für gesundes Bauerntum, die den sogenannten Rusch-Test propagierte (Jurtschitsch 2009: 30–33). Die Spatendiagnose war ein einfaches wie kostengünstiges Verfahren, um die Beschaffenheit des Ackerbodens zu überprüfen und Veränderungen etwa in der Ausprägung der Durchwurzelung und Bodenkrümelung zu beurteilen (Vogt 2000: 293). Die Durchführung des Verfahrens war insofern anspruchslos, als es bei entsprechenden Kenntnissen direkt auf dem Acker durchgeführt werden konnte. Man benötigte dafür nur einen „Flachspaten nebst 2 Stützen, 1 Gärtnerspaten, 1 Kralle, Abdeckbrettchen, 1 Schreibblock und 1 Kamera“ (Preuschen 1981: 4). So ausgerüstet konnten der Berater, der Landwirt oder der Bodenkundler mit wenigen Handgriffen ein Bodenprofil ausheben, vor Ort aufbauen, begutachten und gegebenenfalls dokumentieren. Einige Übung und Detailkenntnisse waren allerdings Voraussetzung. „Man darf nie mit den Händen in das Profil hineingreifen, weil man schon durch leichtes Zusammendrücken die Struktur verändert“, lauteten die Anweisungen der SÖL etwa (Preuschen 1981: 5). Die Wiedereinführung der Spatenmethode war ein kleiner, aber wichtiger Schritt, zurückzugewinnen, was mit solchen einfachen Methoden ebenfalls verloren zu gehen drohte: die Autonomie und Unabhängigkeit des Praktikers und damit die unmittelbare Nähe zu seinem Gegenstand, sprich: des Bauern zur natürlichen Lebensgrundlage, zu Boden, Tier und Pflanze.

Die Spatendiagnose war in den 1970er Jahren nahezu unbekannt und fast in Vergessenheit geraten. Genau genommen handelte es sich nicht einmal um eine Wiederentdeckung. Die SÖL befreite sie nur zum richtigen Zeitpunkt mitsamt der Bodenbiologie, aus der sie hervorgegangen war, aus ihrem Schlummer. Entwickelt hatte sie der im Jahr 1877 geborene Johannes Görbing, der nach einem verschlungenen, aber nicht unüblichen Berufsweg über die Ausbildung als Apotheker, Pharmazie- und Nahrungsmittelchemiestudium, Tätigkeiten in staatlichen Ämtern und Versuchsstationen zur Nahrungsmittelüberwachung und Abwasserreinigung, Kriegseinsatz als Stabsapotheker und Hygieniker schließlich Anfang der 1920er Jahre zur Agrikulturchemie und Bodenkunde fand (Doerell 1948: 103–104; Böhm 1997: 85–86). Görbing, der sich seitdem als „Bodenbiologe“ bezeichnete, verschrieb sich der landwirtschaftlichen Beratung und entwickelte im Laufe der 1920er und 1930er Jahre in seiner privat betriebenen Forschungsanstalt für Bodenkunde und Pflanzenernährung eine „Agrikulturphysiologie“, in deren Zentrum die Fruchtbarkeit des Ackerbodens, deren Diagnose und Erhalt standen (Görbing 1947: 3–9). Neben verschiedenen chemischen Methoden wie elektrometrischer pH-Messung des Bodenzustandes entwickelte er praxistaugliche Verfahren, darunter die „Pflanzen- und Wurzeldiagnose“ und eben die „Spatenmethode“, welche jedem, „auch dem einfachsten Manne“ vor Augen führen können sollten, „wie das, was er getan hat, auf den Acker wirkt“ (Doerell 1948: 104–105). Dabei verstand Görbing die auf biologischen Grundlagen entwickelte Bodendiagnose „in ihrer sorgfältigsten Durchführung und genauesten Handhabung“ nicht bloß als ein Behelfsmittel, sondern auch als eine wissenschaftliche Methode in einem Gebiet, das sich „konventionellen Methoden naturwissenschaftlicher Beschreibung weitgehend entzog“ (Zitat: Uekötter) (Görbing 1947: 18; Uekötter 2010: 88 und 315). Seine Arbeiten fanden nicht nur in der Lebensreform-Bewegung und in der von dieser verehrten Kompost- und Humuswirtschaft breite Aufnahme (Doerell 1948: 104; Vogt 2000: 68–69, 293). Görbings Adepten in der Bodenkunde riefen Anfang der 1940er Jahre mit dem Reichsbodengesundheitsdienst eine Beratungsorganisation ins Leben, deren Mitarbeiter sich als „Hausärzte“ des Bodens und „Bodendiagnostiker“ verstanden und zu deren wichtigsten „ärztlichen“ Instrumenten selbstverständlich der Spaten gehörte (Doerell 1948: 103, 107; Esterházy 1955: 89; Uekötter 2010: 270).

In der Nachkriegszeit bestand das Interesse an der biologisch richtigen Bodenbearbeitung zunächst fort. In der Bundesrepublik und in Österreich entstanden Folgeorganisationen des Bodengesundheitsdienstes, die „das geistige Erbe Görbings im Dienste der Volksernährung“ fortzuführen versuchten (Doerell 1948: 107; Siebert 1948; Bernstorff 1955: 66–67). Letztlich dümpelten sie aber ohne große Reichweite dahin – auch weil die Bodenpflege in Deutschland weiterhin stiefmütterlich behandelt wurde (Uekötter 2010: 270) – und gerieten später unter den Einfluss der Industrie (vgl. Pohl 2001: 284). Diese Entwicklung war bezeichnend vor dem Hintergrund der nachholenden Mechanisierung, die nach langjährigen Konflikten um die Ertüchtigung der Landwirtschaft mit Hilfe von motorisierten Gerätschaften aller Art die Oberhoheit der Industrie über die Äcker sicherte (Uekötter 2010: 317–318). Die Anliegen der Bodenpfleger gerieten damit ins Hintertreffen (vgl. Rid 1984: 328–329).

Auf zunehmend verlorenem Posten kämpfte in jenen Jahren auch Preuschen, der den Bodenbiologen Görbing noch persönlich erlebt hatte und für seinen besonnenen, bodenschonenden Einsatz des neuen landwirtschaftlichen Maschinenparks bewunderte (Preuschen 2002: 211). In seinem 1956 in erster Auflage erschienenen, gemeinverständlichen Buch *Unser Max. Eine Anleitung für den richtigen Schleppereinsatz* erzählte der Max-Planck-Direktor von der fiktiven Bauernfamilie Huber, die nach dem beherzten Kauf eines Traktors allerlei Abenteuer zu bestehen und vor allem die Schwierigkeiten, die das neue Familienmitglied machte, zu meistern hatte. Neben den vielen landwirtschaftlichen, technischen, arbeitsorganisatorischen und auch familiären Zusammenhängen erörterte Preuschen in erzählerischer Form die Probleme der motorisierten Bodenbearbeitung. Früher wusste ein „guter Bauer“, wie sein Boden aussieht und was er zu tun hatte. Wenn er hinter dem Pflug herging, hatte er Zeit genug, den Boden zu beobachten (Preuschen 1956: 68). Doch oben auf dem Traktor konnte man nicht mehr ohne Weiteres einen „gesunden und kranken Boden unterscheiden“ oder sehen, „wie tief man einen Boden und mit welchen Geräten man ihn am besten bearbeitet“. Auf diese behutsame Weise versuchte Preuschen, den Landmännern und Landfrauen die Spatendiagnose ganz im Duktus eines Görbings nahezubringen (vgl. Rheinwald & Preuschen 1956: 3–4, 37–38; Engelien 1948; Uekötter 2010: 76).

Nachdem die Lehren der „biologischen Bodenbearbeitung“ im Zuge der Mechanisierung und Chemisierung der Landwirtschaft in den 1950er und 1960er Jahren weit in den Hintergrund getreten und nahezu vergessen worden waren (Preuschen 1981: 2), kamen die Überlegungen in der SÖL sehr schnell auf die alten Anliegen des biologischen Landbaus zurück. Dabei war die Handschrift der älteren Generation unübersehbar. Preuschen – aber auch der *Spiritus Rector* der SÖL Schumacher – verehrten geradezu das Konzept der Bodenfruchtbarkeit als die einzig wahre nachhaltige Grundlage der Ernährungssicherung in der Welt (Preuschen 1981: 1; Kieffer 1992: 176–178). Als die wichtigsten europäischen landwirtschaftlichen Erfindungen benannte Preuschen nicht umsonst den Räderpflug, die Fruchtfolge und die Düngung mit Stallmist (eine Erfindung des „fränkischen Abendlands“) (Preuschen 1962: 9–12). Die SÖL-Gremien waren sich vor diesem Hintergrund einig, dass sich die Stiftungsarbeit auf den Boden, den Erhalt und die Entwicklung seiner Fruchtbarkeit sowie die „Bodengesundung“ konzentrieren musste (Schaumann et al. 2002: 104–105). Die ökologische Landwirtschaft sollte den Weg zurück zu den alten Werten einer „vernünftigen Humuswirtschaft“ und zur systematischen Entwicklung und generationenüberdauernden (heute: nachhaltigen) Pflege des Kulturbodens weisen. Dazu sollten zunächst die verdrängten Praktiken und scheinbar simplen Landbautechniken wiederbelebt werden. Die Aktivitäten der SÖL reichten bis nach Österreich (Jurtschitsch 2009: 28, 202, 250–251). 1981 erschien in der Schriftenreihe der SÖL eine erste ausführliche Anleitung zur Spatendiagnose unter dem Titel *Die Kontrolle der Bodenfruchtbarkeit *(Preuschen 1981).

Die Pläne der SÖL zur Reaktivierung vergessener Wissensbestände und alter Praktiken folgte einem pragmatischen Kalkül. Die SÖL strebte weder den Einklang mit der Natur an noch naturnahe Bäuerlichkeit und lehnte dementsprechend den Einsatz neuer Technologien und Methoden im modernen Agrarbetrieb nicht rundweg ab. Der Reformeifer des gealterten Agrarberaters Preuschen und anderer Bodenbiologen richtete sich im Namen der Effektivierung der Landarbeit und der Technisierung von Haus und Hof ausdrücklich gegen „falsche Romantik“ und einen gerade im bäuerlichen Umfeld verbreiteten „Traditionalismus“ (Esterházy 1955: 90; Preuschen 1962: 8; Oberkrome 2009: 260). Vielmehr ging es um eine bedachte und selektive Rückbesinnung auf alternative „Landarbeitsformen“, solange diese ihre Nachhaltigkeit historisch unter Beweis gestellt hatten und sich als Vorbild für alternative Wege im modernen Landbau empfahlen (Preuschen 1973: 304–305). Die Wiedereinführung der Spatendiagnose folgte dieser Vorgabe einer abgeklärten Kombination von Altem und Neuem. Sie sollte eines der Instrumentarien sein, Kulturböden in ‚naturgemäßer‘ Weise zu erhalten und zugleich die Bodenfruchtbarkeit zu steigern (vgl. Vogt 2000: 293). Einer ihrer Vorteile lag explizit darin, dass sie mit modernen Laboruntersuchungen kombiniert werden und auf diese Weise helfen konnte, „alte Erfahrungen und neue Erkenntnisse“, das heißt auch biologische Bodenkunde und moderne biologische Forschung zu verbinden (Preuschen 1981).

Mitte der 1980er Jahre initiierte die SÖL ein erstes dreijähriges Forschungsprojekt „Bodenentwicklung“ zur Rekultivierung konventioneller Agrarböden in Zusammenarbeit mit dem Evangelischen Bauernwerk in Baden-Württemberg unter maßgeblicher finanzieller Förderung durch den WWF (*World Wide Fund For Nature*) und der Schweisfurth Stiftung (vgl. Hampl 1988). 1994 schloss sich ein gemeinsam mit der Landesanstalt für Pflanzenbau und Pflanzenschutz in Mainz durchgeführtes und vom Ministerium für Landwirtschaft, Weinbau und Forsten Rheinland-Pfalz gefördertes Forschungsprojekt zur ökologischen Bodenbewirtschaftung (PÖB) an (Beste 2002: 24). Ziel der Untersuchungen war es, die Vergleichbarkeit der Spatenmethode für die wissenschaftliche Dokumentation zu verbessern (Hampl-Mathy 1991: 8; Beste 2002: 4–8). Kritik hatte sich bereits in der Vergangenheit gerade an der einfachen Durchführbarkeit und dem qualitativ-deskriptiven Vorgehen festgemacht. Die Versuche gingen deshalb dahin, das Verfahren auszudifferenzieren und durch quantifizierbare Labormessungen zu ergänzen. Die Praxistauglichkeit der Methode – leicht erlernbar, schnelle Durchführung, Ergebnisse unkompliziert vermittelbar – sollte dabei unbedingt erhalten bleiben. Aus den Untersuchungen ging die „Erweiterte Spatendiagnose“ hervor, die Feldmessungen und Laboruntersuchungen kombinierte und dabei eine Reihe von zusätzlichen Parametern erfasste: systematische Gefügebeurteilung, Aggregatstabilitätstest, Messungen von Bodenfeuchte, Porenvolumen, Lagerungsdichte, Abscherwiderstand und Wurzeldichte (Beste et al. 2001).

An die Stelle der älteren Generation, die den Erfinder der Spatendiagnose noch selbst erlebt hatte, trat damit auch eine jüngere Generation, die zwischen Universität und gemeinnützigem Verein ihren Platz im ökologischen Aufbruch suchte. Das auf zehn Jahre ausgelegte Forschungsprojekt PÖB entwickelte sich geradezu zu einer Verjüngungskur der ökologischen Bodenkunde. 17 mit viel „jugendlicher Energie“ durchgeführte Diplomarbeiten und fünf Dissertationen gingen daraus hervor (Landesanstalt für Pflanzenbau und Pflanzenschutz & Stiftung Ökologie & Landbau (SÖL) 2002: 113–116; Kussel 2002: 109). Hauptbeteiligte an dem Forschungsprojekt waren Ulrich Hampl, Norbert Kussel und Andrea Beste (Preuschen 2002: 351). Hampl war 26 Jahre, als er nach seinem Studium der Agrarwissenschaften an der TU München-Weihenstephan Mitte der 1980er Jahre zur SÖL stieß und dort mit einer Dissertation begann, die er am Institut für Beratungskunde der Landwirtschaftlichen Hochschule Hohenheim abschloss (Preuschen 2002; vgl. Hampl 1995). Anschließend blieb Hampl bei der SÖL und übernahm 1999 die Leitung des Seminarbauernhofs der SÖL in der Südpfalz. Zeitgleich betreute er das Langfrist-Projekt PÖB. Der Landwirt Kussel, der zusammen mit seiner Familie seinen Bauernhof für das Projekt zur Verfügung stellte, übernahm die Betreuung vor Ort auf den mehr als 200 Kilometer von Bad Dürkheim entfernt in der Eifel gelegenen Versuchsfeldern (Hampl 2002: 9). Darüber hinaus beteiligte er sich an der Fortentwicklung verschiedener wissenschaftlicher Verfahren wie etwa zur Erfassung der Mesofauna (vgl. Kussel 2002). Die studierte Geographin Andrea Beste arbeitete ab 1996 im Forschungsprojekt mit und promovierte über die *Weiterentwicklung der Spatendiagnose zu einer wissenschaftlichen Methode zur Bodenbeurteilung *an der Universität Gießen (Beste 2002). Im Anschluss gründete sie mit dem Büro für Bodenschutz und Ökologische Agrarkultur eine private Beratungsstelle und Fortbildungsagentur (Beste 2020). Hampl und Beste führen bis zum heutigen Tag Kurse zur Spatendiagnostik durch.

Wie im Fall von Preuschen und seinem Adepten Hampl trafen Vertreter*innen zweier Generationen in der SÖL aufeinander, die im ökologischen Landbau ihre gemeinsame Aufgabe fanden, deren Lebenswege allerdings einige markante Unterschiede aufwiesen. Hampl hatte vor seinem Landwirtschaftsstudium Zivildienst absolviert und in dieser Zeit am Aufbau des Bundes für Naturschutz in Bayern mitgewirkt (Projektgruppe 2011). Preuschen hatte sich in jungen Jahren dem Nationalsozialismus verschrieben, zunächst als selbstständiger Berater im Osten des Deutschen Reiches gearbeitet, dann als Wissenschaftler und Direktor eines vom nationalsozialistischen Reichslandwirtschaftsministerium für ihn eigens eingerichteten Kaiser-Wilhelm-Instituts beim Aufbau der deutschen Landwirtschaft in den besetzten Gebieten Weißrusslands mitgewirkt (Heim 2003: 91–102; Raehlmann 2005: 127–129; Schwerin: in Vorbereitung). Aus Preuschens Lebenslauf sprach die ganze Hybris, welche die Rückbesinnung auf das Vergangene zu prägen drohte. Politisch-ideologische Konflikte brachen in der SÖL indes wohl deshalb nicht offen aus, weil die Macher*innen das Vergangene im Filter der Sachlichkeit und in Hinblick auf die Zukunft der ökologischen Landwirtschaft reaktivierten. Die deutlichsten Residuen nationalsozialistischen Denkens fanden sich wohl in der gesellschaftlichen Bedeutung, welche ehemalige nationalsozialistische Protagonisten wie Preuschen dem traditionellen Bauerntum zumaßen (vgl. Oberkrome 2009), wobei gerade diese Betonung des Primärsektors entgegen den Trends im gesellschaftlichen Wandel als ein Kernanliegen des zeitgenössischen ökologischen Denkens gesehen werden kann.^16^ Das eindeutig rechtsextreme Milieu jedenfalls, das die Kieffers in Form des Weltbundes zum Schutz des Lebens (WSL) und des Abfall-Bundes (ANS) noch zum erwähnten Strategiegespräch im Jahr 1975 eingeladen hatten (vgl. Geden 1999: 105–117; Köster 2017: 266–267), blieb bei der weiteren Arbeit der SÖL außen vor.

Durch die modernisierungskompatible Mobilisierung der Vergangenheit erlebte die Spatendiagnose in der landwirtschaftlichen Praxis, in der Bodenkunde und im Beratungswesen eine über ihre ursprüngliche Verbreitung hinausgehende Renaissance. Publikationen auf dem neuesten Forschungsstand ersetzten die gemeinverständliche Anleitung von 1979, die die SÖL 1994 in der sechsten Auflage ein letztes Mal herausbrachte (vgl. Markl & Hampl 1996; Beste 2003). Die International Soil Conservation Organisation (ISCO) forderte auf ihrem Treffen in Bonn im Jahr 1996 genau solche, auch für Nichtwissenschaftler*innen anwendbare Methoden der Bodenbeurteilung ein (ISCO 1996: 9). Heutzutage empfehlen Landwirtschaftskammern, Landesämter und Landesanstalten die Spatendiagnose als ein Verfahren zur Untersuchung des Bodengefüges und der Bodenfruchtbarkeit, das inzwischen auch mit der Auswertung genomischer Daten kombinierbar ist (vgl. Dinzen & Buchner 2009; Diez et al. 2017: Titelblatt; Senger 2019; Kreisverwaltung Groß-Gerau 2019).

## Schluss: Vergangenheit und Zukunft des Gegenwissens

Die Geschichte der Stiftung Ökologischer Landbau (SÖL) und der Wiedereinführung der Spatendiagnose als landwirtschaftliche Methode führt vor Augen, wie sich Gegenwissen an der Schnittstelle von Umweltbewegung und akademischer Wissenschaft in den 1970er Jahren formieren konnte. Die Rolle von Zeitlichkeiten, insbesondere von Vergangenheitsbezügen fallen in dieser gegenwissentlichen Formation besonders auf. Die Rückbesinnung auf alte, verdrängte oder einfach vergessene Wissensbestände, Praktiken und Erfahrungen, ihre Reaktivierung und Weiterentwicklung kennzeichneten demnach einen der Wege zum Alternativwissen der Umweltbewegung der 1970er und 1980er Jahre.

1. Abgelegte oder marginalisierte Wissensbestände: Die Wissensinhalte der biologisch ausgerichteten Bodenforschung der 1920er bis 1940er Jahre erlebten im ökologischen Landbau der 1970er Jahre eine Renaissance. Beispielhaft dafür steht die von Johannes Görbing entwickelte „biologische Bodenkunde“ in Theorie und Praxis. In den Lehr- und Forschungsinhalten der Hochschulen waren diese Wissensbestände vor dem Hintergrund der Dominanz der Technologie-getriebenen und Chemie-basierten Intensivlandwirtschaft verloren gegangen. Außerakademische Institutionen, wie die SÖL, machten sich zur Aufgabe, die durch solche „Prozesse des Vergessens“ (Uekötter 2010: 319) erzeugten Wissenslücken auszugleichen.
2. Persönliche Erfahrungsräume: Die Gründungsgeneration der SÖL blickte auf ein erfahrungsreiches Berufsleben zurück. Protagonisten wie der wissenschaftliche Berater der SÖL Gerhardt Preuschen hatten agrarökologisches Wissen und ökologische Landbaupraxis in zurückliegenden Zeiten aus erster Hand erworben. Wie ein Wissensspeicher konnte Preuschen die entstandene Wissenslücke zwischen der selbst erlebten Vergangenheit der „biologischen Bodenkunde“ der 1930er und 1940er Jahre und der Gegenwart der Intensivlandwirtschaft ausfüllen. Bei der Gründungsgeneration handelte es sich zudem in nicht wenigen Fällen um Eliten aus Industrie (Ehepaar Kieffer), Politik (Mansholt) und vor allem der Wissenschaft (Hochschullehrer). Erst in den 1980er Jahren kamen die jungen Vertreter*innen der Umweltbewegung dazu, was sich mit Beobachtungen von Radkau zur Bedeutung sozialer Eliten für die Umweltbewegung allgemein deckt (Radkau 2011: 140–143).
3. Tradierte Praktiken: Vorbilder alternativer landwirtschaftlicher Praxis waren zu Beginn der Renaissance des ökologischen Landbaus in den 1970er Jahren nur vereinzelt vorhanden. Diese hatten über Jahrzehnte am Rande der konventionellen Landwirtschaft überdauert, konnten nun aber wie eine Flaschenpost von der SÖL hervorgezaubert und als Gewährsfälle für die Praktikabilität des ökologischen Landbaus dienen. Zu den tradierten Praktiken gehörte auch die in den 1930er Jahren entstandene ‚ganzheitliche‘ landwirtschaftliche Beratung, die in der Zwischenzeit durch die intensivlandwirtschaftliche Dominanz im Beratungswesen weitgehend in Vergessenheit geraten war und an die die SÖL anknüpfte. Sie sollte dabei helfen, über technische und betriebswirtschaftliche Fragen hinaus alle relevanten Aspekte der Umstellung von konventionellem auf ökologischen Landbau zu berücksichtigen.
4. Vergangenheitsschlaufen als Denkmodus: Der Rekurs auf Vergangenes selbst war keine Erfindung der Protagonist*innen des ökologischen Landbaus in den 1970er Jahren. Schon in früheren Schriften alternativer Landbauwissenschaftler*innen und Praktiker*innen bildete der Bezug auf alte Wissensbestände und Praktiken, die die Technisierung der Landwirtschaft verschüttet hatte, einen wichtigen Ausgangspunkt, wie ein Nachruf auf den Erfinder der Spatendiagnose zeigt: „Görbing war ein besonders lehrreiches Beispiel dafür, wie fruchtbar die Vereinigung alter Weisheit mit neuer Erkenntnis ist“ (Engelien 1948). Der Aufruf, „Wertvolles aus dem Erfahrungsschatz unserer Vorfahren“ (Engelien) zu schöpfen, wurde im Laufe der Geschichte des biologischen und ökologischen Landbaus in dieser Weise als eine epistemische Tugend verstanden und wiederholt eingefordert. Dies korrespondierte mit der Kritik am Modell linearen wissenschaftlich-technologischen Fortschritts, dem die etablierten Wissenschaften Vergangenheits-vergessen folgten. Das „alternative Wissen“ dagegen lief auf eine mehrschichtige, nicht zuletzt zeitliche Öffnung akademischer Wissenserzeugung hinaus. Dies entspricht dem Befund von Vogt, dass die ökologische Landbau-Bewegung in den 1970er Jahren zunächst aus der Adaption alter Erkenntnisse und Techniken schöpfte, in den 1980ern dann sukzessive damit begann, alte Konzepte, Techniken und Praktiken weiterzuentwickeln (Vogt 2000: 285).
5. Konservative Modernisierung: Das Fortschrittsverständnis der Protagonist*innen der SÖL und allgemeiner des ökologischen Landbaus bestand – auch in Abgrenzung zum „bio-dynamischen“ Landbau – darin, einen Ausgleich zwischen wissenschaftlich-technischem Fortschritt und bewusster Rückbesinnung auf die, nicht zuletzt durch diesen Fortschritt verdrängten landwirtschaftlichen Kenntnisse, Praktiken und Strukturen aufzuzeigen (vgl. Vogt 2000: 261). Die Rückbesinnung auf die Erkenntnisse, die die biologische Bodenkunde in der ersten Hälfte des 20. Jahrhunderts hervorgebracht hatte, steht beispielhaft für einen solchen versachlichten Traditionalismus. Dieser erwies sich damit auch als kompatibel mit den vorwärtsdrängenden Experimentierfreuden der jungen Gegenszene, die nicht die Reparatur des naturwissenschaftlich-technischen Fortschritts anstrebte, sondern auf der Suche nach pragmatischem Krisenwissen war, das aus dem Katastrophenkurs der kapitalistischen Gesellschaft raussteuern und Wege zu einer neuen „Gegengesellschaft“ öffnen konnte. Die Trennung zwischen konkretem Wissen und Praktiken auf der einen Seite und den in den Vergangenheitsschlaufen zum Teil mitgelieferten Ideologien auf der anderen Seite mochte allerdings prekär sein. Die Klage über den Verlust traditionellen Bauerntums mochte auf diese Weise immer wieder in eine rückwärtsgewandte Agrarromantik umschlagen und damit auch den Horizont des Utopischen wieder verringern.

In den 1990er Jahren entwickelte sich rund um die Verbesserung und Evaluierung der Spatendiagnose eine eigene Forschungscommunity zwischen Universität und SÖL (vgl. Beste 2002: 8–20). Das stark gewachsene Interesse an den biologischen Bedingungen der Bodenfruchtbarkeit fand auch in der Wissenschaft Widerhall. Die Schnittstelle zwischen Pflanze und Boden, die Rhizosphäre, bildete in diesen Jahren den Gegenstand eines stark aufstrebenden Forschungsgebiets, in dem Mikrobiologie, Bodenkunde und Ökologie miteinander in Beziehung traten (Kickuth 1982; Vogt 2000: 283–285). Umgekehrt, so könnte eine weiterführende These lauten, machte der Brückenschlag zwischen Ökosystem-Theorie und biologischer Bodenkunde es einfacher, die Anliegen des ökologischen Landbaus mit den Interessen der Umweltbewegung politisch miteinander zu verknüpfen.

## Footnotes

1 Vgl. Einleitung zu dieser *Special Section*: Schwerin (2022).

2 Zum Problem der Zeitlichkeit im Überblick siehe Esposito, Fernando: Zeitenwandel. Transformationen geschichtlicher Zeitlichkeit nach dem Boom. Eine Einführung. In: Fernando Esposito (Hg.): *Zeitenwandel. Transformationen geschichtlicher Zeitlichkeit nach dem Boom*. Göttingen: Vandenhoeck & Ruprecht 2017, 7–62.

3 Nach Mende bewegte sich das Denken im Alternativmilieu im Wesentlichen im Rahmen der Gegenwart oder einer begrenzten Zukunft (Mende 2017: 173–177). Es wäre zu fragen, ob der Zeithorizont des gegengesellschaftlichen Aufbruchs der 1970er Jahre differenzierter gesehen werden muss und wann er sich ebenfalls – möglicherweise erst später in den 1980er Jahren – verengte (vgl. Schwerin 2022).

4 Der Begriff konservativer Modernisierung meint hier spezifisch den Versuch bzw. die Programmatik, die technische Entwicklung durch Rekurs auf traditionelle Strukturen (hier: ländliche Gesellschaft) bzw. traditionelle Praktiken und traditionelles Wissen (hier: bäuerlicher Ackerbau und ökologischer Landbau) in gewünschte Bahnen zu lenken.

5 1991 wurden die SÖL mit ihrer Schwesterstiftung Mittlere Technologie zur gemeinnützigen Stiftung Ökologie & Landbau zusammengelegt (Vogt 2000: 85).

6 Zum Rundgespräch eingefunden hatten sich der Theologe Günter Altner (*1936), der Ministerialrat Martin Boesler (*?) vom Hessischen Ministerium für Landwirtschaft und Umwelt, der Agrarwissenschaftler Andreas‑J. Büchting (*1946), der Sozialwissenschaftler und Präsident der deutschen Sektion des Weltbundes zum Schutz des Lebens (WSL) Werner Haverbeck (*1909), der Ingenieur Eduard Schönlein (*?) vom Münchener Arbeitskreis für die Nutzbarmachung von Siedlungsabfällen (ANS), Walter Schwab (*?) vom Pfälzischen Arbeitskreis für naturgemäße Lebensweise, Erich Siefert (*1914) vom Göttinger Arbeitskreis Naturgemäßer Landbau, der Student Dieter Teufel (*1953) vom Bundesverband Bürgerinitiativen Umweltschutz e. V. (BBU) und Ernst Ulrich von Weizsäcker (*1939) (Büchting 2002; Haverbeck: Geden 1999: 112; Siefert 1943; Teufel: Anonym 1980; Weizsäcker 2014: 3).

7 Die Mitglieder im Beirat der SÖL waren bei Gründung bis auf den Biologen und Physiker Prof. Ernst Ulrich von Weizsäcker (*1939), den Botaniker Prof. Kurt Egger (*1932) und den Botaniker Prof. Wolfgang Hagemann (*1929) über 50 Jahre alt: der Physiker Prof. Walter Heitler (*1904), der Unternehmer Max Himmelheber (*1904), der Umwelthygieniker Prof. Karl-Heinz Knoll (*1922), der Landwirtschafts-Politiker Dr. Sicco L. Mansholt (*1908), der Physiologe Prof. Hans Schaefer (*1906), der Ökonom Dr. Ernst Friedrich Schumacher (*1911), der Hydrologe Dr. Gerhard Helmut Schwabe (*1910) und der Botaniker Prof. Heinrich Zoller (*1923). (Zu Heitler: Rasche & Thellung 1982; Knoll 1998: 5; Schaefer: Klee 2005: 523–524; Hagemann: Drüll 2009: 247–248; Mansholt: Merriënboer 2011; Himmelheber & Sauer 2016; Egger: Lünzer 1992: 370; Zoller: Erhardt 2010; Schumacher: Varma 2003: 122; Schwabe: Mollenhauer & Hickel 1989).

8 Graf (2008: 215–217); Mende (2011: 300–301); vgl. auch Kieffer (1982: 69–71); Sturm (1980). Die *Scheideweg*-Gruppe lag damit auf einer Linie mit dem frisch gewählten Vorsitzenden des Bundes für Umwelt- und Naturschutz Deutschland (BUND) und (Noch‑)CDU-Bundestagsabgeordneten Herbert Gruhl sowie dem Schriftsteller und späteren Präsidenten der E.-F.-Schumacher-Gesellschaft Carl Amery.

9 1990 zog die Geschäftsstelle nach Bad Dürkheim um (Vogt 2000: 85).

10 Die Lebensreform- und Siedlungsbewegung der Weimarer Republik verfolgte im Zusammenhang ihres besonderen Interesses für gesunde Ernährung auch neue Wege in der Landwirtschaft. In den Landbauwissenschaften gab es vielfältige Bestrebungen, den „natürlichen Landbau“ gegen die Übermacht der Agrikulturchemie zu stärken, mit dem Ergebnis, dass schon damals biologische Landbaumethoden neu begründet und noch ältere wiederentdeckt wurden (Vogt 2000: 49; Uekötter 2010: 214–242).

11 Seit den 1970er Jahren erschien die Buchreihe *Ökologische Konzepte*, die vierteljährlich publizierte Zeitschrift *Ökologie & Landbau* und die Schriftenreihe *SÖL-Sonderausgaben* zu aktuellen Themen in Theorie und Praxis. Ab den 1990er Jahren erschien der *Beraterrundbrief für den ökologischen Landbau* und unter Beteiligung der SÖL *Der kritische Agrarbericht*, ab 2000 die Buchedition *Praxis des Ökolandbaus* (Schaumann et al. 2002: 98–103).

12 Im Jahr 1982 wirtschaften 700 der 800.000 Betriebe in der Bundesrepublik alternativ auf 13.000 ha landwirtschaftlicher Nutzfläche (Rid 1984: 287). Preuschen war auch in Österreich aktiv (Jurtschitsch 2009: 28).

13 Solche Erwartungen weckten leicht Befürchtungen vor einer bevorstehenden „Ökodiktatur“ (Vierhaus 1994: 28–38).

14 Diese Art der Kritik bestimmte Preuschens praktische und wissenschaftliche Tätigkeit, seit er in den 1930er Jahren dafür gestritten hatte, die Motorisierung der Landwirtschaft behutsam in den Gesamtzusammenhang der landwirtschaftlichen Arbeit und Erhaltung der Bodenfruchtbarkeit einzupassen (Preuschen 2002: 281; Raehlmann 2005: 128). Zur Technokratiedebatte in der Bundesrepublik siehe Stehr (1994: 420–426).

15 Umgekehrt traf Preuschen im Umfeld des Ökolandbaus auf „das nachdenkliche Milieu“, das er in der Intensivlandwirtschaft der Nachkriegszeit „so schmerzlich vermisst hatte“ (Uekötter 2010: 419).

16 Persönlich hegte Preuschen allerdings noch im hohen Alter Sympathien für die Zeit des Nationalsozialismus (vgl. Preuschen 2002: 199–245).
